# Commentary: Treating Diseases from Alzheimer’s to Parkinson’s Using Transcranial Pulse Stimulation: Mechanistic Insights, Recent Evidence, and Ethical Considerations

**DOI:** 10.3390/neurosci6020056

**Published:** 2025-06-17

**Authors:** Lars Wojtecki

**Affiliations:** 1 Institute of Clinical Neuroscience and Medical Psychology, Medical Faculty, Heinrich Heine University, 40225 Düsseldorf, Germany; wojtecki@uni-duesseldorf.de; 2 Department of Neurology and Neurorehabilitation, Hospital Zum Heiligen Geist, Academic Teaching Hospital of the Heinrich-Heine-University Düsseldorf, 47906 Kempen, Germany

**Keywords:** Alzheimer’s disease, Parkinson’s disease, transcranial pulse stimulation, mechanotransduction, ethics, neuromodulation, randomized controlled trials

## Abstract

Transcranial pulse stimulation (TPS) is a non-invasive neuromodulation method that uses, high-intensity acoustic shockwaves to deliver focused mechanical stimulation to neural tissue with minimal thermal effects. The mechanism of action includes but is not limited to promotion of blood flow and angiogenesis through mechanotransduction. Clinical data to date are limited and preliminary. In Alzheimer’s disease (AD), TPS has demonstrated cognitive and mood improvements in pilot studies and secondary endpoint analysis in first randomized trials. The enhancement of gamma-band oscillations and network connectivity has been reported. Clinical observations in Parkinson’s disease (PD) suggest TPS as a hypothesis-generating approach to address non-motor symptoms—such as depression, cognitive decline, and the freezing of gait—through theoretical modulation of basal ganglia–cortical circuits. TPS is CE-marked in Europe for AD and shows a favorable safety profile; however, ethical considerations arise from the limited evidence base, potential impairment of patient autonomy and judgment in dementia, and the risk of withholding established treatments. TPS should only be offered under structured scientific protocols or within patient registries to ensure rigorous oversight. Ensuring that consent processes account for cognitive capacity, and that TPS is applied as adjunct rather than replacement therapy, is paramount. Future research must include large-scale randomized controlled trials (RCTs), standardize stimulation protocols, deepen mechanistic insight, and embed robust ethical frameworks.

## 1. Introduction

Non-invasive brain stimulation (NiBS) techniques have become central to dementia research, particularly in Alzheimer’s disease (AD), where mounting evidence suggests they can enhance cognition or slow decline across disease stages [1,2]. Transcranial pulse stimulation (TPS) delivers highly focused shockwave pulses guided by neuronavigation, enabling precise targeting beyond the superficial cortex and offering a novel adjunctive modality for AD management [3,4]. In Parkinson’s disease (PD), pharmacological treatments such as L-Dopa effectively address motor symptoms, yet non-motor features remain challenging to treat. This context highlights the hypothesis-generating potential of TPS to ameliorate non-motor symptoms, warranting dedicated investigation.

Given the evolving evidence base and the vulnerability of dementia populations, early ethical considerations—such as ensuring patient autonomy, robust informed consent procedures, and avoiding premature replacement of established therapies—must be integrated from the outset. TPS should be offered only under structured scientific protocols or within registries to safeguard participant welfare and promote equitable access.

## 2. Mechanism of Action

Transcranial pulse stimulation delivers brief, high-pressure acoustic shockwaves that mechanically deform neuronal and vascular membranes, activating mechanosensitive ion channels and presumably triggering intracellular cascades [4,5,6]. This mechanotransduction probably leads to upregulation of neurotrophic factors (e.g., BDNF, GDNF) and vascular mediators (VEGF, nitric oxide), promoting neuroplasticity and angiogenesis [7,8]. EEG and fMRI studies demonstrate enhanced gamma-band (~40 Hz) coherence and restoration of functional connectivity within hippocampal–cortical and salience networks—changes correlated with improved memory and mood [9,10,11]. Unlike continuous ultrasound modalities, TPS’s single-pulse approach minimizes energy absorption and heating, yielding highly focal, non-thermal neuromodulation.

## 3. Recent Clinical Evidence in Alzheimer’s Disease

Across early-phase trials that provide preliminary data, TPS has shown moderate to large effects on cognition and mood (Cohen’s d ≈ 0.5–0.8 for cognitive domains and up to d ≈ 1.4 for neuropsychiatric symptoms [1,2,3,12,13].

In a recent randomized, sham-controlled clinical trial published by Matt et al. involving 60 participants TPS demonstrated significant secondary endpoint improvements in executive function and depression in younger patients under the age of 70 years, although the primary cognitive endpoint was narrowly missed—likely due to insufficient washout in the crossover design. Importantly, cognitive assessments were primarily based on the CERAD battery [14].

In contrast, Radjenovic et al. reported outcomes from a retrospective cohort of 58 dementia patients demonstrating significant improvements in elderly patients with the best responders’ mean age being 73 years [15]. Our findings also suggest that older patients and those at more advanced disease stages might benefit from TPS [16]. Beyond cognitive outcomes, imaging studies provide additional support for TPS-induced brain changes. Popescu et al. demonstrated reduced cortical atrophy in TPS-treated AD patients using MRI morphometry [17], while Dörl et al. found selective functional connectivity normalization in fMRI analyses after TPS [18]. These results suggest that TPS may induce structural and network-level brain adaptations underlying clinical benefits. These findings suggest that TPS may be beneficial across different disease stages, though large-scale randomized controlled trials (RCTs) are needed.

## 4. Safety Profile

TPS has been administered in thousands of sessions across AD, PD, and psychiatric indications with a favorable safety record. Mild side effects—headache, scalp discomfort, transient fatigue—occur in approximately 4–6% of sessions and resolve without intervention [13,14,15,16]. No serious device-related adverse events, such as seizures or hemorrhages, have been reported; post-treatment MRI and histology confirm absence of thermal damage or microcavitation [4,6,9]. Standard TPS parameters (0.2–0.25 mJ/mm^2^; 6000 pulses) optimize tolerability, but long-term registry data are imperative to detect rare or delayed effects.

## 5. Ethical Considerations in Neurodegenerative Disease

Neurodegeneration impairs judgment and consent capacity; AD patients may lack insight, necessitating formal capacity assessments and representative involvement. Neuromodulation may alter personality—adversely or by restoring pre-morbid traits—so consent must address cognitive, emotional, and identity changes. TPS, approved for AD only, should only be offered under structured scientific protocols or within patient registries with robust safety monitoring. TPS to date cannot be regarded as standard of care. TPS is investigational, and ethical considerations must prioritize patient safety above access to unproven interventions. However, ethical discussions also need to address the problem that withholding TPS from eligible patients may also raise ethical concerns given its regulatory approval and its good safety profile that is available so far. For patient registries, transparent benefit–risk discussions need to ensure that TPS complements rather than replaces guideline treatments. Off-label neuroenhancement is ethically untenable without evidence. In Table 1, our team outlines a checklist for an ethical framework for further discussion.

## 6. Patient Selection, Preparation, and Protocols

Patient selection should follow the CE-marked indication for Alzheimer’s disease, with diagnosis confirmed by clinical criteria and CSF biomarkers. Patients remain on their standard-of-care regimens (e.g., cholinesterase inhibitors, antipsychotics) throughout TPS.

Exclusion criteria should include the following:

- Intracerebral pathologies such as tumors, hematoma, or infections unrelated to AD;
- Vascular lesions corresponding to Fazekas grade 3 white-matter changes;
- Cerebral amyloid angiopathy;
- Recent anti-amyloid antibody therapy due to ARIA risk;
- Coagulopathies;
- Corticosteroid use;
- Seizure disorders;
- Non-MRI-compatible implants;
- Severe behavioral disturbances;
- Pregnancy;
- Other factors impacting therapy adherence.

Informed consent should involve capacity assessment and, when appropriate, a legally authorized representative. Standardized cognitive and behavioral assessments should be conducted at baseline and post-treatment using parallel test forms in a distraction-free environment. While an initial course of six sessions over two weeks is standard in AD protocols, the consideration of monthly booster sessions may help sustain clinical benefits over the long term. Moreover, although current targeting emphasizes cortical areas relevant to Alzheimer’s (e.g., frontal, temporal, parietal, precuneus), the exploratory application of TPS to other regions—such as the supplementary motor area or premotor cortex—could be investigated in future studies for alternative indications.

## 7. Preliminary Findings in Parkinson’s Disease

Pilot investigations in PD have primarily targeted motor symptoms but are extremely limited and mainly hypothesis-generating to date. Osou et al.’s retrospective series (*n* ≈ 20) reported UPDRS-III motor improvements following open label daily TPS over two weeks [19]. Manganotti et al. observed acute resting tremor reductions after a single session in nine patients under sham-controlled conditions [20]. These early findings stand in contrast to highly effective dopaminergic therapies for motor control. Thus, therapeutic equivalence between TPS and standard Parkinson’s treatments cannot be concluded. However, a perspective therapeutic niche may lie in ameliorating mood, cognition, and gait initiation. This has to be addressed in future research. Robust, double-blind, sham-controlled trials with comprehensive non-motor and functional mobility endpoints are lacking and should be prioritized.

## 8. Conclusions and Future Directions

This commentary aims to give an overview of the current evidence of TPS in neurodegenerative diseases, and furthermore, our team proposes a checklist for ethical framework for further discussion.

TPS is CE-marked in Europe for AD and presents a favorable safety profile; nonetheless, ethical concerns regarding limited evidence, impaired patient autonomy warrant structured protocols, large-scale, randomized controlled trials (RCTs) and registries. TPS should be only adjunctive to standard therapy under rigorous oversight. Future research should standardize protocols, deepen mechanistic insights, and embed ethical safeguards. An important practical consideration is the potential contraindication of TPS in patients receiving anti-amyloid antibody therapies (such as lecanemab, aducanumab, or donanemab). Since these therapies carry a risk of ARIA (amyloid-related imaging abnormalities) and associated cerebral microbleeds, mechanical stimulation with TPS may potentially exacerbate such risks. Careful patient selection and rigorous MRI monitoring should be applied when considering TPS in populations exposed to monoclonal antibody treatment. Until robust safety data are available, concurrent use of TPS and antibody therapy should be approached with great caution or avoided.

When it comes to PD, while there are positive aspects for motor and possibly non-motor function to advance clinical translation, the field must conduct multicenter, randomized, placebo-controlled trials with standardized dosing, stratified cohorts, adequate washout designs, and long-term safety registries. Therapeutic equivalence between TPS and standard Parkinson’s treatments cannot be concluded. However, a perspective therapeutic niche may lie in ameliorating mood, cognition, and gait initiation.

Beyond neurodegeneration, TPS is under exploration for major depression [21], mild cognitive impairment [22], ADHD [23], autism spectrum disorder [24], post-stroke rehabilitation, and chronic pain; rigorous trials are essential.

## Figures and Tables

**Table 1:** Proposed checklist for an ethical framework in TPS.

Ethical Criterion	Explanation
Regulatory Approval	CE-marked in EU for AD; additional country-specific approvals required.
Positive Evidence	Clinical studies should demonstrate high-level evidence with positive outcomes.
Clinical Effect Size	Effect sizes (Cohen’s d) should be comparable to or exceed guideline therapies.
Expert Recommendations	Endorsement or conditional support by recognized expert panels.
Guideline Support	TPS use should align with existing professional treatment guidelines.
Best-Evidence NIBS Method	Preference given to non-invasive methods with the strongest supporting evidence.
Refractory Disease	Consideration for patients who have not responded adequately to standard treatments.
Preserve Standard Therapy	No approved standard treatment should be withheld or replaced solely by TPS application.
Risk Assessment	Risks must be predictable, reasonable, and fully explained to patients.
Scientific Monitoring	Treatments should occur under structured research protocols with ongoing safety monitoring and registries.
Informed Consent	Thorough disclosure of benefits, limitations, and alternatives, including capacity assessments.
Patient Sovereignty	Special attention to cognitive capacity and shared decision-making, recognizing potential personality effects.
Reimbursement Mechanisms	Insurance coverage is desirable but not mandatory, minimizing out-of-pocket expenses and promoting access.
